# Physical activity and happiness of college students: chain mediating role of exercise attitude and sleep quality

**DOI:** 10.3389/fpubh.2025.1544194

**Published:** 2025-01-22

**Authors:** Kehan Li, Roxana Dev Omar Dev, Wenhao Li

**Affiliations:** Faculty of Educational Studies, Department of Sports Studies, Universiti Putra Malaysia, Serdang, Malaysia

**Keywords:** physical activity, exercise attitude, sleep quality, happiness, college students

## Abstract

**Introduction:**

This study explores the role of physical activity, exercise attitude, and sleep quality in college students’ happiness, aiming to provide a theoretical basis for improving college students’ mental health.

**Methods:**

The study employed a whole group sampling method, utilizing a physical activity level scale, exercise attitude scale, sleep quality scale, and happiness scale. A questionnaire survey was conducted with 1,308 college students from four universities in China.

**Results:**

The analysis yielded three key findings: (1) Physical activity significantly improves college students’ happiness, and this improvement can be achieved through direct and indirect effects. (2) Positive exercise attitude plays an important mediating role between physical activity and happiness, and a high level of exercise attitude can enhance happiness. In addition, the effect of physical activity on happiness is most significant through exercise attitude. (3) Although sleep quality did not significantly mediate the relationship between physical activity and happiness alone, it played a positive role in the chain mediation path of “physical activity → exercise attitude → sleep quality → happiness.”

**Discussion:**

The discovery of the chain mediation path shows that physical activity not only affects happiness, but also indirectly affects college students’ happiness through the combined effects of psychological and physiological factors.

## Introduction

Happiness, as a fundamental aspect of human experience, has been a central focus of philosophy and religion for centuries, with influential figures such as Aristotle, Confucius, and Buddha emphasizing its role in defining a fulfilling life (1). In contemporary research, subjective happiness is conceptualized as a multidimensional construct, encompassing life satisfaction, emotional states (e.g., happiness, sadness, anger, stress, and pain), and a sense of purpose and meaning in life (2). Empirical evidence suggests that subjective happiness is significantly associated with both physical health and age, with physical health challenges and the aging process often contributing to a decline in happiness levels (2). Moreover, mental health emerges as a critical determinant of subjective happiness, with a positive correlation reported between happiness and mental health status (3).

In the Chinese context, recent data highlights the mental health vulnerabilities of youth. The China National Mental Health Development Report (2021–2022), published in 2023, identifies individuals aged 18–24 as a high-risk group for depression, with a prevalence rate of 24.1%, significantly higher than other age groups. Notably, half of those affected within this demographic are students (4). Consequently, enhancing subjective happiness among college students has been proposed as a strategic intervention to improve their mental health outcomes (5). Physical activity represents a promising avenue for enhancing subjective happiness. Setting realistic goals and achieving them through physical exercise has been shown to improve self-confidence and intrinsic satisfaction, thereby positively influencing happiness levels (6). The relationship between physical activity, mental health, and happiness is well-documented, with interventions involving low-to-moderate intensity aerobic exercise demonstrating efficacy in improving both mental health and happiness among college students over several weeks (7). Conversely, reduced levels of happiness have been associated with negative outcomes, including poorer sleep quality, diminished physical health, impaired academic performance, and increased susceptibility to mental health issues, all of which may contribute to declines in academic achievement (8).

Building on the resilience framework, Rutter’s two-factor model of problem behavior underscores the importance of examining both risk factors that exacerbate maladaptive outcomes and protective factors that mitigate such risks (9). Within this context, physical activity emerges as a protective factor, offering a pathway to enhance happiness. This study aims to investigate the psychological mechanisms through which physical activity impacts happiness, with a specific focus on integrating attitudes toward exercise and sleep quality. By examining these interactions, the research seeks to provide both theoretical and practical insights into reducing depressive symptoms and promoting happiness among college students.

### Physical activity and happiness

Physical activity constitutes a fundamental and significant aspect of daily life, encompassing various activities associated with study and work. Activities such as sitting and walking are also considered forms of physical activity. The American College of Sports Medicine (ACSM) defines physical activity as any bodily movement generated by skeletal muscle contraction that results in energy expenditure exceeding the basal metabolic rate (10). A widely accepted definition proposed by Caspersen describes physical activity as any bodily movement induced by skeletal muscle contraction that leads to energy metabolism (11).

Happiness is influenced by a multitude of factors, among which physical activity has increasingly drawn the attention of psychologists (12). As a core component of positive mental health, happiness can be enhanced through physical exercise, which has been shown to improve resilience against emotional fluctuations (13). Herbert reported that physical activity and sports interventions are frequently employed to alleviate perceived stress, reduce mental health symptoms, and enhance both psychological and physiological dimensions of happiness among college students (7). Regular physical activity demonstrates a positive correlation with happiness and overall physical and mental quality of life. A meta-analysis conducted by Hale revealed that, among adolescents aged 11–19, mental health and happiness indices improved proportionally with increased physical activity levels, while depression indices decreased accordingly (14). Similarly, Kliziene observed in an eight-month intervention study that engaging in two hours of physical activity per week facilitated progress toward physical health-related goals, enhanced happiness, and promoted the adoption of a healthy lifestyle (15).

Guo and Cui emphasized that sedentary individuals, such as college students and office workers, should cultivate the habit of participating in physical activity to address muscle atrophy resulting from insufficient exercise (16). This adaptation can enhance physical health and thereby improve subjective happiness. A systematic literature review by Zhang and Chen highlighted that even minimal physical activity, such as 10 min per week or exercising once weekly, can significantly boost happiness (12). Both aerobic exercise and stretching or balance activities have been shown to effectively enhance happiness, with a consistently positive correlation observed between physical exercise and happiness. Furthermore, Diener and Tay posited that physical activity may serve as a critical factor in understanding happiness, suggesting that investigating the relationship between physical activity and happiness represents a promising research domain (17).

### Different physical activity levels and happiness

The International Physical Activity Questionnaire (IPAQ), designated by the World Health Organization in 1998, underwent extensive testing for reliability and validity across 12 countries and 14 regions by the year 2000, and has since been recommended for global application. Hashimoto developed a physical activity scale, later revised by Li, categorizing physical activity into three levels: high, moderate, and low (18, 19). Hale demonstrated that engaging in an average of 60 min of moderate to high-intensity physical activity per week significantly enhances happiness (14). Similarly, Moljord reported in a cross-sectional study that students participating in moderate-intensity exercise 2 to 3 times per week exhibited significantly higher happiness scores compared to those engaging in low-intensity exercise once per week or less (20). Interestingly, no significant difference in happiness was observed between youth exercising 2 to 3 times per week and those engaging in high-intensity exercise nearly every day.

In contrast, Mohammadi highlighted that students undertaking high-intensity physical activity weekly demonstrated statistically higher happiness levels compared to those engaging in moderate-intensity activity (21). Furthermore, Hosker identified that moderate to high-intensity aerobic exercise, performed 3 to 5 days per week for 45 to 60 min per session, significantly benefits the physical and mental health of youth, particularly by improving their happiness index (22).

This growing body of evidence underscores the critical role of physical activity in enhancing happiness, reinforcing the importance of exercise attitude and its impact on sleep quality and overall happiness.

*H1:* Physical activity has a significant positive predictive effect on college students’ happiness.

### The mediating role of exercise attitude

Huang proposed that physical exercise attitude is a critical psychological component influencing individuals’ participation in sports activities (23). It is an acquired, relatively stable, and enduring cognitive, emotional, and behavioral intention toward sports. A positive exercise attitude plays a crucial role in fostering the development of lifelong physical activity. Exercise attitude is identified as a primary psychological factor that affects sports behavior (24). Wilson demonstrated in their study that exercise attitude mediates the relationship between psychological needs and self-achievement motivation (25). Markland also found that implicit exercise attitudes can be enhanced through image-based interventions targeting exercise intention, which subsequently improve individuals’ positive emotions and overall happiness (26). Similarly, Zhang and Yang (27) suggested that physical exercise has the potential to improve college students’ happiness and personality traits, indicating a positive correlation between exercise attitude and happiness.

Cheon and Lim (28) in their longitudinal study, concluded that exercise attitude significantly influences the level of physical activity. Their findings suggest that participation in sports activities can reduce stress and positively impact youth’ happiness. Moreover, one of the most important factors shaping individuals’ beliefs, attitudes, and behaviors is the pursuit of meaning, defined as the effort to acquire accurate and consistent information (29). There is a well-documented positive relationship between life meaning, life goals, and attitudes toward sports and exercise (30, 31). Gatab and Pirhayti (32) observed that different types of sports influence male college students’ attitudes toward exercise and their levels of happiness. Specifically, male students participating in group sports exhibited stronger exercise attitudes and higher happiness levels compared to those engaging in individual sports.

Research utilizing structural equation modeling (SEM) has revealed significant relationships between exercise attitude, happiness, and loneliness. The standardized path coefficient for the positive relationship between exercise attitude and happiness is 0.10, while the negative correlation between exercise attitude and loneliness is −0.20. These findings suggest that improvements in youth’ positive attitudes toward sports are associated with increased happiness and decreased loneliness.

In recent years, scholars have increasingly examined the relationship between physical activity and exercise attitude. According to Mao, physical exercise attitude encompasses eight dimensions: behavioral attitude, goal attitude, behavioral cognition, behavioral habits, behavioral intention, emotional experience, behavioral control, and subjective standards (33).These dimensions collectively provide a psychological perspective on individuals’ participation in physical activity and the factors shaping their exercise attitudes. Notably, attitudes exist in both explicit and implicit forms (34). Padin emphasized that even when controlling for explicit attitudes, implicit exercise attitudes independently predict subsequent physical activity levels (35).

Kjønniksen in a ten-year longitudinal study, demonstrated that exercise attitudes during youth have long-term effects on physical activity levels. Most youths exhibit positive exercise attitudes, which significantly influence their engagement in lifelong physical activities (36). Zhang highlighted that moderate and high-intensity physical activities improve exercise attitudes in both young men and women (37). Among the dimensions of exercise attitude, “behavioral habits,” “behavioral intentions,” and “behavioral control” show significant positive correlations with the number of students who pass the third-level basketball test. Additionally, Graham found that youths with more positive attitudes toward sports, exercise, and fitness engage in 30–40% more moderate-to-vigorous physical activity per week compared to their peers with less positive attitudes (38). These differences persisted over time, with youths participating in 2.1 additional hours of activity per week after five years and 1.2 h per week after ten years. Notably, happiness has a moderating effect on exercise attitude, and exercise attitude can reliably predict physical activity levels.

*H2:* Exercise attitude plays a mediating role between college students’ physical activity and happiness.

### The mediating role of sleep quality

Sleep as a fundamental physiological activity, has been extensively studied across various disciplines, particularly in physiological and psychological medicine (39). Traditionally, sleep is conceptualized as a generalized inhibitory process, where this inhibition spreads from the cerebral cortex to the brain’s subcortical centers, ultimately inducing a state of sleep (40). Despite extensive research, there remains no universally accepted definition of sleep quality within academic discourse. Sleep quality is typically assessed using subjective and objective approaches. Subjective measures rely on self-reported questionnaires or sleep diaries, capturing individual experiences, while objective measures employ instruments such as sleep polygraphs and electroencephalograms, providing precise data on sleep parameters (41).

Empirical evidence underscores the critical role of sleep quality in happiness. Bardosono in a cross-sectional study, identified sleep quality as the primary determinant of happiness in children, surpassing even dietary influences (42). Similarly, Novak and Lev-Ari demonstrated that prolonged stress negatively affects happiness, with sleep quality mediating this relationship (43). Otsuka analyzed data from 64,329 youth, revealing a linear relationship between sleep disturbances and subjective happiness scores, further supported by dose–response correlations identified through multivariate logistic regression (44). In adults, short sleep duration and poor sleep quality have been associated with reduced happiness (45). However, Konjarski showed that happiness is negatively correlated with sleep duration (46). Therefore, some researchers believe that more research is needed to explore the relationship between sleep duration and happiness and consider factors such as age or other variables (47). Furthermore, Badri (48) observed that older adults are more susceptible to sleep disorders, which are independently and closely associated with declines in subjective happiness. Collectively, these findings highlight the robust connection between poor sleep quality and diminished happiness, underscoring the critical role of sleep in promoting health and happiness (49, 50)^.^

Sleep is a cyclic and transient functional state regulated primarily by neurobiological processes. Increasing evidence suggests that factors such as nutrition, physical activity, and sleep hygiene significantly influence sleep quality (51). For instance, Bisson (52) reported that long-term physical exercise positively impacts sleep quality, with even moderate activity levels improving sleep outcomes. In a clinical study involving 129 adults with obstructive sleep apnea (OSA), regular physical exercise was shown to reduce daytime drowsiness, enhance peak oxygen intake, and improve overall sleep quality (53). Additionally, Fairbrother examined 91 youth aged 11–19 and found that 73.6% experienced difficulty maintaining sleep, while 60.5% reported trouble falling asleep (54). Adherence to physical activity guidelines—60 min of moderate to vigorous activity daily—was associated with improved sleep quality and reduced sleep latency in this cohort.Shen conducted a survey of 416 youth (215 men and 201 women) and found that physical exercise and insomnia (*r* = −0.268, *p* < 0.01) were positively correlated (55). Promoting youth to participate in physical exercise can help alleviate insomnia problems and improve the quality of sleep for youth.

Contrasting evidence exists regarding the relationship between physical activity and sleep quality. For instance, Zhao and Yi (56) in a meta-analysis, found no significant effect of physical activity on sleep quality improvement (*p* = 0.15). Similarly, Memon (57) in a meta-analysis of college students, reported inconsistent associations between physical activity and sleep, noting a weak negative correlation between high-intensity physical activity and sleep duration within a random-effects model. Despite these inconsistencies, physical activity is hypothesized to enhance sleep quality through mechanisms such as increased adenosine levels and body temperature regulation. However, the timing of physical activity remains contentious, as evening exercise may elevate physiological arousal and disrupt sleep (51).

Happiness has consistently been shown to correlate positively with sleep quality. However, the association between sleep quality and physical activity levels remains inconclusive, warranting further exploration.

*H3:* Sleep quality mediates the relationship between physical activity and happiness among college students.

### The mediating role of exercise attitude and sleep quality

Attitude as a Determinant of Behavior: Insights into the Relationship between Exercise Attitude and Sleep Quality.

Attitude is a precursor to behavior, often shaping individuals’ actions and cognitive processes. Despite its importance, research directly linking exercise attitude to sleep quality remains limited. Most studies explore exercise attitude as a mediating variable within the broader context of physical activity or exercise. Existing evidence underscores the significant positive effects of regular physical activity on sleep quality, with exercise attitude serving as a critical mediator in this relationship.

Firstly, substantial research highlights the direct impact of physical activity on sleep quality. For instance, Gulia and Kumar (58) demonstrated that consistent exercise improves sleep duration, enhances sleep efficiency, and alleviates symptoms of insomnia and other sleep disorders. Similarly, aerobic exercise has been shown to mitigate symptoms of sleep apnea and facilitate deeper sleep stages (59). These findings suggest that regular physical activity exerts physiological benefits, such as enhancing deep sleep and reducing nighttime awakenings.

Secondly, the relationship between exercise attitude and sleep quality has garnered increasing scholarly attention. A positive attitude toward exercise has been shown to enhance individuals’ exercise self-efficacy, thereby fostering adherence to regular physical activity. Williams and French reported that individuals with favorable exercise attitudes are more likely to maintain consistent exercise routines, indirectly contributing to improved sleep quality (60). Additionally, a positive exercise attitude has been associated with greater psychological satisfaction and reduced stress levels, factors that significantly promote high-quality sleep (61).

Furthermore, research has suggested that exercise attitude may also function as a moderating variable in the exercise-sleep relationship. Jackson and Dishman observed that exercise attitude influences not only individuals’ participation in exercise but also the intensity and frequency of their physical activity (62). These factors, in turn, determine the extent to which exercise improves sleep quality. Therefore, cultivating a positive attitude toward exercise may represent a critical strategy for enhancing sleep quality.

In summary, exercise attitude serves as both a mediating and moderating variable in the intricate relationship between physical activity and sleep quality. Promoting a positive exercise attitude may provide a valuable approach to improving overall sleep health and happiness.

*H4:*: exercise attitude and sleep quality play a chain mediating role between physical activity and happiness.

### Research hypothesis

**Figure 1:**
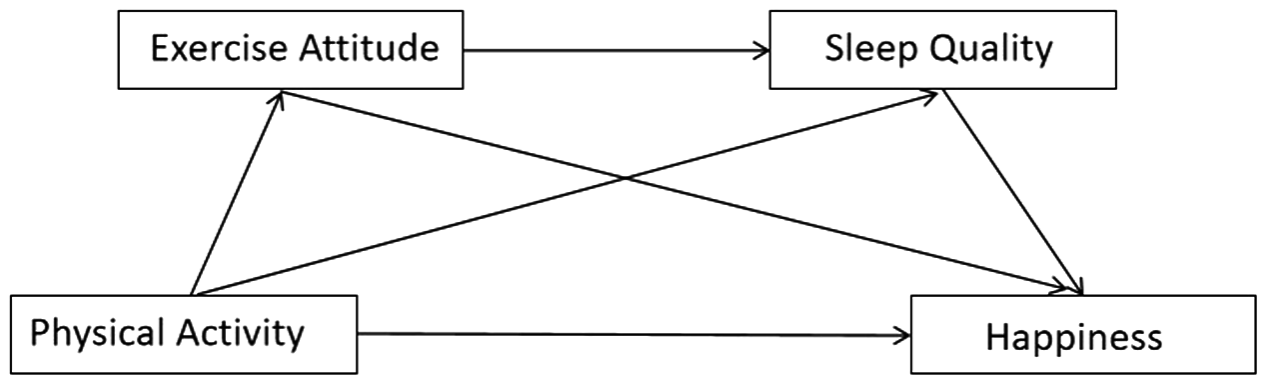
The hypothetical structure model.

In summary, in order to further explore the intrinsic relationship between physical activity and happiness, this study aims to construct a chain mediation model as shown in Figure 1 and verify the following aspects:

(1) Physical activity can positively predict college students’ happiness.
(2) Exercise attitude plays an independent mediating role between physical activity and college students’ happiness.
(3) Sleep quality plays an independent mediating role between physical activity and college students’ happiness.
(4) Exercise attitude and sleep quality play a chain mediating role between college students’ physical activity and happiness.

### Data source and method

#### Research subjects

This study employed a class-based whole group sampling method to select participants. A total of 1,308 college students, ranging in age from 18 to 24 years and spanning freshman to senior years, were recruited as research subjects. The participants were drawn from multiple academic disciplines and majors at four higher education institutions: Sichuan University of Arts and Sciences, Chongqing College of Mobile Communication, Henan Normal University, and Daqing Normal University, located in Sichuan, Chongqing, Henan, and Heilongjiang provinces and municipalities. Detailed demographic and institutional information is provided in Table 1.

**Table 1:** Information of the subjects.

Sort	Category	Number of people	Percent (%)
Total quantity	—	1,308	100
Gender	Male	606	46.3
	Female	702	53.7
Subject background	Humanities and social sciences	506	38.7
	Natural sciences	576	44
	Physical education	226	17.3
Grade	Freshman year	654	50
	Sophomore year	458	35
	Junior year	141	10.8
	Senior year	55	4.2
Place of origin	Town	973	74.4
	City	335	25.6

The inclusion criteria for participation were as follows: (1) Participants were full-time college students, either from undergraduate programs or vocational colleges, with the exception of psychology majors; (2) Participants had no history of mental illness, demonstrated clear logical thinking, and were capable of independently completing the questionnaire; (3) All participants voluntarily consented to partake in the study; (4) Informed consent forms were distributed by counselors or class teachers, and participants commenced the survey only after completing and signing these forms; (5) Participants were in good physical health, with individuals who were ill or hospitalized excluded from the study.

### Survey procedure

To ensure the effectiveness of the questionnaire survey, the research staff provided respondents with a detailed explanation of the purpose, content, and methods of the research in a face-to-face format. They also addressed all queries raised by the participants to enhance their understanding and willingness to participate. With consent from the students and their counselors (or class teachers), participants completed the questionnaires anonymously. Personal information, such as student names and identification numbers, was not collected to protect their privacy and ensure confidentiality. The questionnaires were distributed electronically through the “Questionnaire Star” platform, with links shared via class WeChat groups or by scanning QR codes provided on-site. Completing the questionnaire required approximately 10 min.

Of the 1,450 individuals invited to participate, 1,378 completed and submitted the questionnaire, yielding a response rate of approximately 95%. To ensure data accuracy and enhance the stability of the structural equation model, rigorous screening was conducted by the first author. Invalid responses were excluded, including submissions completed in less than five minutes, responses with uniform answers for at least 10 consecutive questions (e.g., “1,111,111” or “2,222,222”), and patterned answers following repetitive rules (e.g., “5, 4, 3, 2, 1, 5, 4, 3, 2, 1” or “5, 5, 5, 4, 4, 4, 3, 3, 2, 2, 2, 1, 1, 1”). After this process, 1,308 valid questionnaires were retained, achieving a validity rate of 94%.

To express gratitude for their participation, respondents were offered small tokens of appreciation, such as keychains or vouchers. Based on local legal requirements, this study did not require ethical committee review or signed informed consent. However, to ensure ethical rigor, participants were provided with detailed information about the survey on the first page of the electronic questionnaire. This included the purpose, methods, and process of the survey, as well as assurances that the data would remain anonymous, be used solely for scientific research, and pose no risk to participants’ daily lives. Participation was entirely voluntary, and respondents could proceed only after confirming their informed consent.

### Instruments

#### Physical activity level scale

The International Physical Activity Questionnaire (IPAQ), developed by the World Health Organization in 1998, was validated for reliability and validity across 12 countries and 14 regions by 2000 (63). This study utilized the IPAQ-Short Form (IPAQ-S), which comprises seven items. Six of these assess physical activity levels, encompassing weekly frequency (days per week) and daily cumulative time (minutes per day) for activities of varying intensities. Activities lasting fewer than 10 min are excluded from the calculation. Sedentary behavior was not considered in this study.

The MET (metabolic equivalent of task) values applied in IPAQ-S are as follows: walking (3.3 METs), moderate-intensity activities (4.0 METs), and vigorous-intensity activities (8.0 METs). Weekly physical activity levels are calculated as:

The total physical activity level is derived by summing the MET values for all three intensity levels. Criteria for categorizing high, moderate, and low physical activity levels are detailed in Table 2.

**Table 2:** Criteria for judging physical activity levels.

Grouping	Standards
High	Meet any one of the following two criteria:
	1. All kinds of high-intensity physical activities total ≥ 3 days, and the total weekly physical activity level ≥ 1,500 MET-min/w per week
	2. Three intensities of physical activity in total ≥ 7 days, and the total weekly physical activity level is greater than or equal to 3,000 MET-min/w
Middle	Meet any one of the following three criteria:
	1. Meet at least 20 min of various high-intensity physical activities per day, totaling ≥3 days
	2. At least 30 min of various moderate-intensity and walking activities per day for a total of ≥5 days
	3. A total of 3 intensities of physical activity ≥5 days, and the total weekly physical activity level ≥ 600 MET-min/w
Low	Meet any one of the following two criteria:
	1. No activity was reported
	2. Some activity is reported but does not meet the medium and high grouping criteria

In this study, the IPAQ-S demonstrated acceptable reliability and validity, with a Cronbach’s alpha coefficient of 0.73. The confirmatory factor analysis indices further supported its applicability for the sample: *χ*^2^/df = 4.049, GFI = 0.994, AGFI = 0.976, IFI = 0.991, TLI = 0.971, CFI = 0.992, and RMSEA = 0.048. These results align with the recommendations of Macfarlane confirming the scale’s suitability for the study (64).

### Exercise attitude scale

The scale utilized in this study was developed by Mao to measure attitudes toward physical exercise (33). It comprises 70 items distributed across eight dimensions: behavioral attitude, goal attitude, behavioral cognition, behavioral habits, behavioral intention, emotional experience, behavioral control, and subjective standards. The scale includes both positively and negatively scored items. Higher overall scores reflect a more positive attitude toward physical exercise. Responses are measured using a 5-point Likert scale, with options ranging from “strongly agree” to “strongly disagree,” scored as 5, 4, 3, 2, and 1, respectively. The total possible score ranges from 70 to 350.

In this study, the scale demonstrated a Cronbach’s alpha coefficient of 0.87, indicating good internal consistency. Confirmatory factor analysis yielded the following fit indices: *χ^2^*/df = 4.626, GFI = 0.996, AGFI = 0.968, IFI = 0.998, TLI = 0.987, CFI = 0.998, and RMSEA = 0.053. These values suggest that the scale has robust reliability and validity. Based on Mao recommendations, the scale is considered appropriate for the sampled population (33).

### Pittsburgh sleep quality index (PSQI)

The Pittsburgh Sleep Quality Index (PSQI) was developed by Buysse to assess sleep quality over the past month (65). Liu translated this scale into Chinese (66). The PSQI consists of 19 self-rated items and 5 observer-rated items, with 18 items included in the scoring process. These 18 items are grouped into seven components: subjective sleep quality, sleep latency, sleep duration, sleep efficiency, sleep disturbances, use of sleep medication, and daytime dysfunction. Each component is scored on a scale ranging from 0 to 3, and the sum of these component scores constitutes the PSQI total score, which ranges from 0 to 21. Higher PSQI scores indicate poorer sleep quality.

Based on the PSQI scores, sleep quality is categorized into three levels: a total score greater than 7 indicates poor sleep quality and potential sleep problems, a score of 4 or lower represents good sleep quality, and scores between these two thresholds indicate moderate sleep quality. Since higher scores on the PSQI indicate poorer sleep quality, whereas the other three scales used in this study reflect more positive outcomes with higher scores, a score transformation was applied to the PSQI to maintain consistency in correlational and mediation analyses. Specifically, the transformation converted scores as follows: 0 was converted to 21, 1 to 20, …, 20 to 1, and 21 to 0.

In this study, the Cronbach’s alpha for the PSQI was 0.84, indicating good internal consistency. The confirmatory factor analysis (CFA) results demonstrated excellent model fit, with the following indices: *χ*^2^/df = 4.269, GFI = 0.992, AGFI = 0.975, IFI = 0.985, TLI = 0.965, CFI = 0.985, and RMSEA = 0.05. These results suggest that the scale possesses robust reliability and validity. Following the recommendations of Ma and Yang the PSQI is deemed suitable for the sample used in this study (67, 68).

### Overall happiness scale

The Overall Happiness Scale was initially proposed by Fazio as a standardized tool for the National Center for Health Statistics in the United States to evaluate individuals’ self-reported happiness (69). The scale was later adapted into a Chinese version by Duan (70). This version consists of 18 items across six dimensions: satisfaction and interest in life, concern about health, energy, depression or happiness, control of emotional behavior, and relaxation versus tension.

Items 1 to 14 are rated on either a 5-point or 6-point Likert scale, while items 15 and 16 use a 10-point scale. Negative items include 1, 3, 6, 7, 9, 11, 13, 15, and 16. In this study, the scale achieved a Cronbach’s alpha coefficient of 0.80. Confirmatory factor analysis produced fit indices of *χ*^2^/df = 4.597, GFI = 0.996, AGFI = 0.975, IFI = 0.996, TLI = 0.981, CFI = 0.996, and RMSEA = 0.052, affirming the scale’s reliability and validity. Following the guidelines of Wang, the scale is deemed suitable for the sample in this study (71).

### Statistical analysis

Statistical analyses were conducted using SPSS 24. Descriptive statistics, reliability tests, one-way ANOVA, and Pearson correlation analysis were applied to the study variables: physical activity, exercise attitude, sleep quality, and happiness. Additionally, PROCESS Model 6 in SPSS was employed to examine the chain mediation effect among these variables.

Structural equation modeling (SEM) was performed using AMOS 24 to fit the chain mediation model and evaluate its fit indices. The mediating roles of exercise attitude and sleep quality between physical activity and happiness were analyzed. Subsequently, a standardized hierarchical multiple regression analysis was conducted. The bias-corrected nonparametric percentile Bootstrap method, with 5,000 resamples, was applied to test the significance of the mediation effects.

Following established guidelines Liu (72), the model’s fit was deemed acceptable if *χ*^2^/df was less than 5, and if GFI, AGFI, IFI, TLI, and CFI were all greater than 0.8, with RMSEA below 0.08.

### Chain mediation model

The chain mediation model is an important statistical analysis method that can deeply explore the complex indirect mechanism between independent variables and dependent variables. Compared with a single mediation model, the chain mediation model can reveal the hierarchical relationship and synergy between multiple mediating variables, thereby more comprehensively explaining the dynamic relationship between variables (73, 74). In this study, the chain mediation model was used to examine the impact path of physical activity on college students’ happiness through exercise attitude and sleep quality, aiming to build a more complex and comprehensive theoretical framework.

The chain mediation model has significant flexibility and accuracy and is particularly suitable for analyzing complex relationships between multiple variables. By combining the path analysis method, researchers can accurately estimate the mediating effect of each indirect path and provide a robust confidence interval (75, 76). Through this comprehensive analysis method, this study can reveal the full picture of the impact of physical activity on happiness.

## Results

### Common method bias test

The Harman single factor test was used to conduct factor analysis on all the items involved in this study (77). It was determined whether the data had systematic errors due to the same acquisition method or measurement environment. According to the results, exploratory factor analysis extracted 18 factors with eigenvalues greater than 1. The explanation rate of the first factor was 28.269%, which was less than the detection standard of 40%. Therefore, it is often considered that there is no common method bias in the data of this study.

### Study on the differences in exercise attitude, sleep quality and happiness between different levels of physical activity

By taking low, medium and high physical activity as independent variables. And taking exercise attitude, sleep quality and happiness as dependent variables, a one-way ANOVA was performed. The variance homogeneity of exercise attitude, sleep quality and happiness was 0.102, 0.143 and 0.335, respectively. The *p* values were all greater than 0.05, satisfying the variance homogeneity, and a one-way ANOVA could be performed (see Table 3). There are significant differences in exercise attitude and happiness levels among the three groups of low, medium and high physical activity. It is positively distributed and the higher the activity level, the stronger the exercise attitude and the higher the happiness. There is no significant difference between different levels of physical activity and sleep quality.

**Table 3:** Analysis of differences in various variables among different physical activity groups (x ± s).

Physical activity	*n*	Exercise attitude	Sleep quality	Happiness
Low	170	217.23 ± 36.849	15.11 ± 2.656	75.65 ± 12.279
Medium	414	233.7 ± 36.691	15.13 ± 2.538	77.22 ± 11.102
High	724	262.8 ± 41.811	15.23 ± 2.666	79.89 ± 12.477
F		128.692**	0.275	11.957**
Partial η^2^		0**	0.759	0**

*p < 0.05; **P < 0.001, the same below.

### Analysis of the relationship between physical activity, exercise attitude, sleep quality and happiness

After Pearson correlation analysis, the results show (see Table 4). Except for physical activity and sleep quality, there are correlations between the other variables and they are all positively correlated. Physical activity is positively correlated with exercise attitude and happiness (*r* = 0.401, *p* < 0.01; *r* = 0.133, *p* < 0.01). There is no correlation between physical activity and sleep quality (*r* = 0.02, *p* > 0.01). Exercise attitude is positively correlated with sleep quality and happiness (*r* = 0.146, *p* < 0.01; *r* = 0.301, *p* < 0.01). Sleep quality is positively correlated with happiness (*r* = 0.474, *p* < 0.01). Happiness is positively correlated with physical activity, exercise attitude and sleep quality. Although there is no correlation between physical activity and sleep quality, exercise attitude can predict physical activity level (28). There is a positive correlation between exercise attitude and sleep quality. There is a significant positive correlation between happiness and sleep quality. Therefore, it shows that the mediation effect can be further tested.

**Table 4:** Means, standard deviations and correlations between variables.

Variable	*M*	SD	1	2	3	4
1. Physical activity	2.42	0.71	1			
2. Exercise attitude	247.67	43.327	0.401**	1		
3. Sleep quality	15.18	2.623	0.02	0.146**	1	
4. Happiness	78.49	12.133	0.133**	0.301**	0.474**	1

*N = 1,308, so the tests are all double tests.*

### Physical activity and happiness: chain mediation test

Based on Wen approach to testing mediation effects, the present study utilized a chain mediation model to examine the relationships among the variables (78). Specifically, physical activity was treated as the independent variable, happiness as the dependent variable, and exercise attitude and sleep quality as mediating variables. Mediation analysis was conducted, and the results are presented in Table 5. Physical activity was found to significantly and positively predict college students’ happiness (*β* = 0.035, *p* < 0.01), thereby supporting Hypothesis 1. Moreover, physical activity significantly and positively predicted exercise attitude (*β* = 0.4011, *p* < 0.01) and significantly and negatively predicted sleep quality (*β* = −0.0464, *p* < 0.01). Exercise attitude significantly and positively predicted sleep quality (*β* = 0.1647, *p* < 0.01) as well as happiness (*β* = 0.2221, *p* < 0.01). Sleep quality also significantly and positively predicted happiness (*β* = 0.4406, *p* < 0.01).

**Table 5:** Regression analysis of variable relationships.

Variable	Equation 1: (Dependent variable: exercise attitude)			Equation 2: (Dependent variable: sleep quality)			Equation 3: (Dependent variable: happiness)		
	*β*	SE	*t*	*β*	SE	*t*	*β*	SE	*t*
Physical activity	0.4011	1.5462	15.8243**	−0.0464	0.1103	−1.5529**	0.035	0.4386	1.3629**
Exercise attitude				0.1647	0.0018	5.5144**	0.2221	0.0073	8.56**
Sleep quality							0.4406	0.11	18.5335**
*R* ^2^	0.1609			0.0231			0.2801		
*F*	250.4092**			15.463**			169.131**		

**p < 0.01.

To further analyze the mediating effects along the indirect effect paths, the bias-corrected percentile bootstrap method (with 5,000 resamples) was employed to estimate the confidence intervals. The findings, presented in Table 6, reveal that the total standardized mediating effect of exercise attitude and sleep quality between physical activity and happiness was 0.0978. Among the three indirect effect paths, the Bootstrap 95% confidence intervals indicated that two paths were significant, as their intervals did not include zero, while one path was not significant. The details of these indirect effects are as follows:

a) The effect of the “physical activity → exercise attitude → happiness” path was 0.0891, accounting for 91.1% of the total effect. The Bootstrap 95% confidence interval for this path did not include zero, indicating significance and confirming Hypothesis 2.
b) The effect of the “physical activity → sleep quality → happiness” path was −0.0204, accounting for −20.86% of the total effect. As the Bootstrap 95% confidence interval for this path included zero, it was not significant, and thus Hypothesis 3 was not supported. Notably, sleep quality did not mediate the relationship between exercise attitude and happiness.
c) The effect of the “physical activity → exercise attitude → sleep quality → happiness” path was 0.0291, accounting for 29.75% of the total effect. The Bootstrap 95% confidence interval for this path did not include zero, indicating significance and supporting Hypothesis 4.

**Table 6:** Analysis of mediation effects between variables.

Effect type	Path	Effect size	BootSE	Boot95%CI		Proportion%
				Lower limit	Upper limit	
Direct effect	Physical activity → happiness	0.1328	0.0274	0.0789	0.1866	----
Indirect effects	Physical activity → exercise attitude → happiness	0.0891	0.0128	0.0643	0.1152	91.10%
	Physical activity → sleep quality → happiness	−0.0204	0.013	−0.0461	0.0053	−20.86%
	Physical activity → exercise attitude → sleep quality → happiness	0.0291	0.0059	0.018	0.041	29.75%
Total indirect effect		0.0978	0.0182	0.0634	0.134	100%

**Figure 2:**
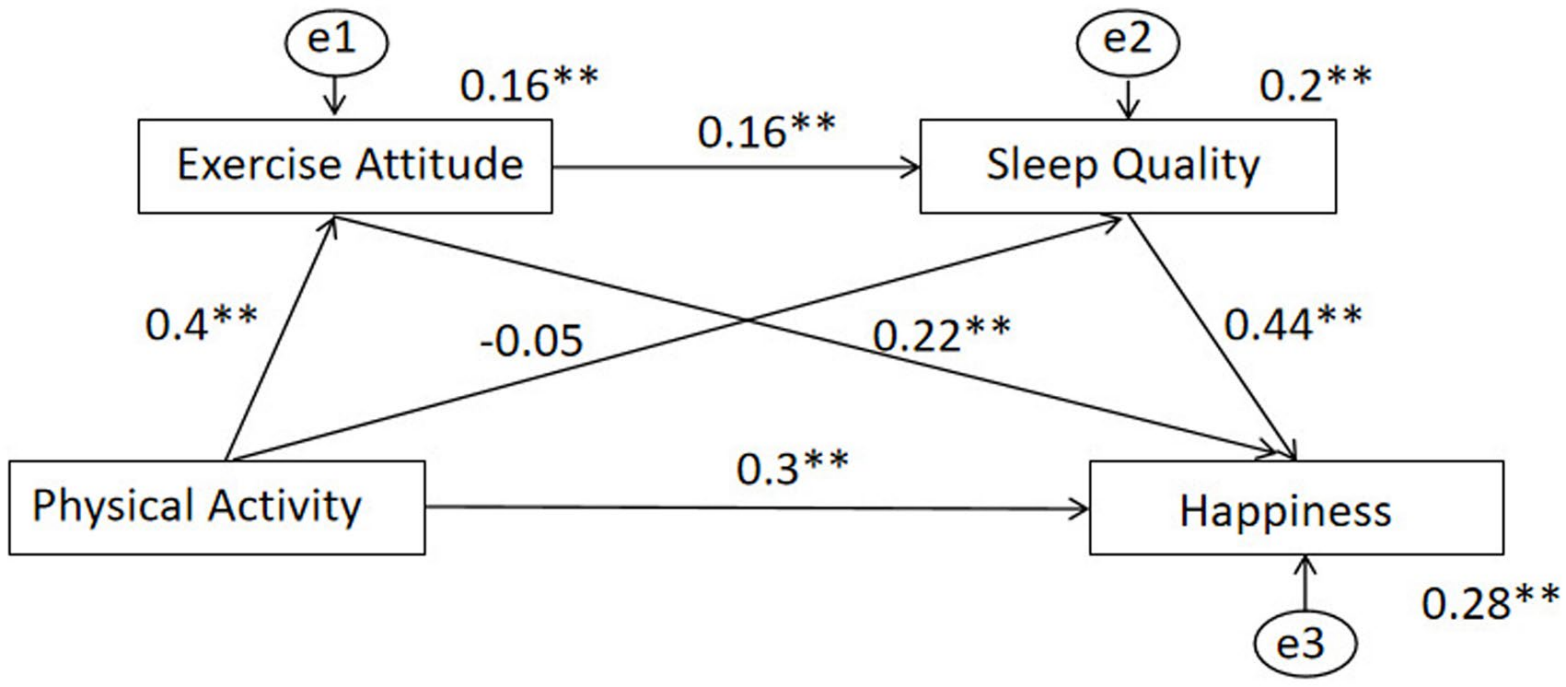
Chain mediation model of exercise attitude and sleep quality between physical activity and happiness ps:**p < 0.001.

These results demonstrate that physical activity indirectly predicts happiness through both the independent mediating effect of exercise attitude and the chain mediating effect of exercise attitude and sleep quality. Among these mediating mechanisms, the independent mediating effect of exercise attitude contributed the largest proportion to the total indirect effect (91.1%). The chain mediation effect of exercise attitude and sleep quality between physical activity and happiness is illustrated in Figure 2.

## Discussion

### Relationship between physical activity and happiness

This study examined the significant positive correlation between physical activity and the happiness of college students, a finding supported by prior research (21, 79, 80). Hypothesis 1, which posits that physical activity plays a vital role in improving the happiness index, was also verified (12, 81). Consistent with previous findings, physical activity significantly enhances the happiness of college students (82), potentially due to the stimulation of endorphin secretion, which promotes positive emotions and mental health (83).

For example, Mohammadi (21) surveyed 156 college students (53 males and 103 females) and found that students engaging in higher levels of physical activity reported greater happiness. This study also observed no significant gender differences in the relationship between physical activity and happiness, aligning with the findings of Zhao (84) who analyzed data from 1,020 Chinese college students, Zhao concluded that physical activity predicts happiness, noting that male students exhibited both higher activity levels and happiness compared to females. Similarly, Chen through a study of 300 college students, confirmed a significant positive correlation between physical activity and happiness, with a moderating effect of gender (85). These studies show that improving the physical activity level of college students can effectively improve their happiness index. The differences between men and women mentioned in these studies need further research.

### The mediating role of exercise attitude

This study also identified exercise attitude as a partial mediator between physical activity and happiness, consistent with existing literature. Hypothesis 2, which highlights the role of exercise attitude, was verified. Exercise attitude refers to an individual’s positive emotions and behavioral tendencies toward engaging in physical activities Ajzen and serves as a crucial predictor of the frequency and persistence of such activities (86, 87). A more positive exercise attitude enables college students to derive greater psychological pleasure and a sense of accomplishment from physical activities, thereby enhancing their happiness. This conclusion aligns with the findings of O’Connor who argued that exercise attitude fosters identification with sports and the development of exercise habits, ultimately contributing to improved happiness (88).

For instance, Compare conducted a study involving 300 women and found that those with positive exercise attitudes scored higher on happiness, indicating that they derived greater emotional satisfaction and psychological benefits from physical activity (89). Similarly, Meng (90) emphasized that attitude predicts behavior, suggesting that the level of physical activity depends on an individual’s attitude toward participation, which in turn correlates with happiness levels. A study involving 800 college students demonstrated that physical exercise significantly improves happiness (90).

These findings underscore the importance of cultivating positive exercise attitudes among students. Colleges and universities are encouraged to design interventions focusing on psychological guidance and behavioral motivation strategies to help students establish long-term exercise habits. By fostering a positive exercise attitude, institutions can promote greater participation in sports activities and, consequently, enhance students’ mental health and happiness.

### The mediating role of sleep quality

In this study, the mediating role of sleep quality between physical activity and happiness was not verified, and hypothesis 3 was not established. This may be because although physical activity can improve sleep by enhancing fatigue and regulating melatonin levels,the impact of sleep quality may be regulated by a variety of complex factors, such as individual living habits, learning pressure, and biological clock (91, 92). Uchida mentioned in their study that physical activity in the afternoon will significantly increase the third and fourth stages of slow wave sleep (SWS) during the next sleep period (93). If exercise is performed in the evening, the amount of SWS is between the “after no exercise” and “afternoon exercise” conditions. And short-term exercise before bedtime can also produce a stress effect, thereby reducing the amount of subsequent SWS. This study shows that exercise at noon can improve sleep, but exercise in the evening or short-term exercise before bedtime has less effect on sleep. According to a national sleep survey by Hirshkowitz about 45% of college students have poor sleep quality at least three days a week, which significantly affects their emotional management, learning efficiency and happiness (92). Although physical activity can promote the sleep quality of college students to a certain extent, if individuals have problems such as frequent late nights and irregular sleep time, the positive effect of physical activity on sleep may be weakened, and the sense of happiness may also be affected. Memon proposed in their study that college students have a variety of lifestyles (57). When college students leave home, they have increased independence and reduced supervision, increased social activities (such as attending concerts, bars, social and sports activities), and often lead to the formation of unhealthy behaviors such as smoking and alcoholism. Compared with these, the positive effects of sports are minimal, so no significant mediating effect can be formed between physical activity and happiness. Xiao added anxiety and mobile phone dependence as mediating variables when studying physical activity and sleep disorders (94). It was found that physical activity not only has a direct negative impact on sleep disorders, but also indirectly predicts sleep disorders through its impact on anxiety and mobile phone dependence.

In summary, this study failed to verify the mediating role of sleep quality, which may be due to the fact that sleep quality is affected by multiple factors, resulting in an unstable effect on happiness. Future studies can attempt to further explore the long-term impact of physical activity on sleep quality and its complex pathways of action on happiness by controlling variables such as lifestyle habits and work and rest schedules.

### Mediating role of exercise attitude and sleep quality

In this study, it was verified that exercise attitude and sleep quality have a chain mediation between physical activity and happiness, and hypothesis 4 was established. Although sleep quality is not correlated with physical activity, it is correlated with exercise attitude (*r* = 0.146, *p* < 0.01) and happiness (*r* = 0.474, *p* < 0.01). Exercise attitude can predict physical activity level (28). When individuals have a positive attitude toward exercise, they are more likely to participate in more intense or regular physical activities. This positive exercise behavior can promote higher quality sleep, thereby improving the individual’s subjective happiness (95). The discovery of this chain mediation path shows that it is not enough to simply encourage students to participate in physical activities. Colleges and universities need to maximize the positive impact of physical activities on students’ mental health by cultivating positive exercise attitudes and sleep management (96).

### Practical significance

This study explored the relationship between physical activity and college students’ happiness, as well as the mediating role of exercise attitude and sleep quality between the two. The results showed that physical activity not only directly and positively predicted happiness, but also indirectly and positively predicted happiness through the independent mediating role of exercise attitude and the chain mediating role of exercise attitude and sleep quality. Based on the results of this study and existing literature, colleges and universities should comprehensively consider the interaction between physical activity, exercise attitude and sleep quality when designing intervention programs. First, campus sports activities such as fitness classes and sports competitions can help students develop a positive attitude toward exercise (97). Second, special sleep management courses can be set up to teach students how to improve sleep quality through regular work and rest and exercise (98). Finally, it is possible to consider introducing psychological counseling programs to help students improve their self-efficacy and enhance their exercise motivation, thereby effectively promoting students’ overall happiness (95, 99).

## Limitations and prospects

First, this study adopted a cross-sectional design. Although it revealed the influence between variables, it was unable to infer the causal relationship between variables. In future research, a longitudinal design can be adopted, which will be more helpful to look at the problem from the perspective of development time. This study found some valuable results, but there are also certain research defects: sampling bias. The questionnaire was distributed by class sampling, which resulted in a gender bias in the sample, that is, fewer males and more females. This sampling bias may affect the external effects of the research results. In addition, some scholars believe that there are gender differences in the impact of physical activity on happiness. This study did not conduct separate analysis on male and female college students. Future research will expand the scope of the survey and examine the relationship between physical activity and happiness.

## Conclusion

(1) Physical activity can significantly negatively and positively predict college students’ exercise attitude and happiness. Physical activity may help improve college students’ exercise attitude and happiness. Sleep quality is positively correlated with exercise attitude and happiness, but has no correlation with physical activity. (2) Physical activity can not only directly predict college students’ happiness, but also indirectly predict college students’ happiness through the independent mediating effect of exercise attitude. And indirectly predict depression through the chain mediation effect of exercise attitude and sleep quality. It is suggested that the interaction of physical activity, exercise attitude and sleep quality should be considered comprehensively when improving and intervening in the happiness of college students.

## Data Availability

The raw data supporting the conclusions of this article will be made available by the authors, without undue reservation.
